# A transcriptomic approach to characterize human monocyte‐derived microglia‐like cells as a potential tool for identifying biological markers of mild cognitive impairment

**DOI:** 10.1002/alz70861_107968

**Published:** 2025-12-23

**Authors:** Marcela Valdés‐Tovar, Ruth Alcalá‐Lozano, Airam Campos, Denise Gómez‐Bautista, Leonardo Ortíz‐López, Jesús Argueta, Héctor Solís‐Chagoyán, Germán O. López‐Riquelme, Edith A. Fernández‐Figueroa, Linda Nelly Patiño, Rocío A. Chávez‐Santoscoy, Silvia A. Hinojosa‐Álvarez

**Affiliations:** ^1^ Instituto Nacional de Psiquiatría Ramón de la Fuente Muñiz, Mexico City, DF Mexico; ^2^ CINCCO‐Universidad Autónoma del Estado de Morelos, Cuernavaca, MR Mexico; ^3^ Instituto Nacional de Medicina Genómica, Mexico City, DF Mexico; ^4^ Instituto Tecnológico y de Estudios Superiores de Monterrey, Monterrey, NL Mexico

## Abstract

**Background:**

Microglial cells are critical in Alzheimer’s disease (AD) pathophysiology. Most human studies have analyzed microglia from postmortem brain samples or microglia derived from induced‐pluripotent stem cells. Our study provides evidence to support human monocyte‐derived microglia‐like cells as a cellular model to identify potential biological markers of mild cognitive impairment (MCI).

**Method:**

Peripheral blood monocytes were isolated from cognitively healthy volunteers (*n* =13, age 50‐75 years old), patients with MCI (*n* =4, age 58‐75 years old), and one patient with possible AD (77 years old). Monocytes were cultured with IL‐34 and GM‐CSF for 14 days to induce the microglial‐like phenotype, which was verified through flow cytometry and immunofluorescence. Total RNA from monocytes and microglia‐like cells was extracted and processed for RNASeq.

**Result:**

From a total of 19549 genes with measured expression, 4648 genes were differentially expressed (DE) in microglia‐like cells (*p* ‐value 0.05; log fold change 2.2). After Bonferroni correction, 26 biological pathways (KEGG), 256 biological processes, 47 cellular components, and 50 molecular functions (Gene Ontology: GO) were significantly impacted. 92% of DE genes from 7 types of monocytes were significantly downregulated, while 54% of DE genes from 3 adult microglial types were significantly upregulated. SPP1, APOE, and TREM2 genes, all three considered AD‐associated genes expressed in brain microglia, were among the top 30 DE genes. When samples from MCI patients were compared with those of cognitively healthy subjects, 262 DE genes were identified, two biological pathways were impacted, and 139 GO‐terms were significantly enriched.

**Conclusion:**

The transcriptomic profile of human monocyte‐derived microglia‐like cells suggests that this cellular model might be suitable for multi‐omic analysis to identify potential biological markers of mild cognitive impairment and AD.

